# Enumeration of the Public Health Workforce in New York State: Workforce Changes in the Wake of COVID-19

**DOI:** 10.3390/ijerph192013592

**Published:** 2022-10-20

**Authors:** Isaac Michaels, Sylvia Pirani, Molly Fleming, Mayela M. Arana, Emily D’Angelo, Cristina Dyer-Drobnack, Margaret DiManno, Sarah Ravenhall, Christian T. Gloria

**Affiliations:** 1 Department of Epidemiology and Biostatistics, University at Albany School of Public Health, Rensselaer, NY 12144, USA; 2 Region 2 Public Health Training Center (PHTC), New York, NY 10032, USA; 3 New York State Association of County Health Officials (NYSACHO), Albany, NY 12110, USA; 4 Department of Sociomedical Sciences, Columbia University Mailman School of Public Health, New York, NY 10032, USA

**Keywords:** public health practice, local health departments, governmental public health workforce, COVID-19 response, public health systems, workforce development

## Abstract

The governmental public health workforce in the United States has faced staffing shortages for over a decade that have been exacerbated by the COVID-19 pandemic. To assess this critical issue, the Region 2 Public Health Training Center collaborated with the New York State Association of County Health Officials to enumerate the city and county public health workforce in New York State. The organizations used an online survey to: (1) count employees and full-time equivalent (FTEs) staff in local health departments in 2021; (2) assess workforce trends since the COVID-19 pandemic; and, (3) identify challenges local health departments encounter in recruiting and retaining qualified public health workers. To assess trends, findings were compared with secondary data from 2019. Despite playing a central role in COVID-19 mitigation, local health departments experienced no overall increase in staffing in 2021 compared to 2019, with many health departments experiencing large increases in vacant positions. Recruitment challenges include noncompetitive salaries, difficulties finding qualified candidates, and lengthy hiring processes. This study complements accumulating evidence indicating that long-term investment in local public health infrastructure is needed to bolster the workforce and ensure that communities are protected from current and future health threats.

## 1. Introduction

A robust local public health workforce is an essential component of any effective public health system. A strong public health workforce is representative of the community it serves, is sufficiently sized to meet the public’s needs, and has the knowledge, skills, and resources to respond quickly to emerging health threats [1]. However, while the U.S. population increased by about 8% between 2008 to 2019, funding reductions decreased among full-time staff in local health departments (LHDs) nationally by roughly 16% [2]. Issues of insufficient public health staffing globally have also been described. A recent Lancet report found that the global health workforce fell short by approximately 43 million in 2019 [3]. Inadequate staffing leaves LHDs neither able to address routine public health issues in their communities, nor able to respond effectively to emergent public health issues such as COVID-19 [4]. LHDs in New York State (NYS) were on the front line of responding to the COVID-19 pandemic. NYS was the epicenter of the pandemic in the U.S., with 48,496 COVID-19 deaths recorded among NYS residents during 2020 and 2021 cumulatively [5]. The pandemic’s impact on NYS and its public health workforce has been significant.

New York’s LHDs promote and protect the health of the more than twenty million people who live in its 62 counties, including one local health department that serves the 8.3 million residents of the five counties (boroughs) that comprise New York City (NYC) and one in each of the other counties outside of NYC. In each jurisdiction, the LHD workforce provides one or more of six core services: community health assessment; communicable disease control; chronic disease prevention; family health services; emergency preparedness; and, in full-service health departments, environmental health services. Costs that LHDs incur by providing these six services are partially reimbursed by NYS’s General Public Health Work Program, established in Article 6 of the state’s Public Health Law [6,7,8]. Article 6 funding reductions, a state-imposed property tax cap that limits local spending, and a recession, have all impacted New York’s LHDs’ abilities in the past ten years to maintain an adequately staffed workforce. Furthermore, because data on the size and composition of New York’s LHD workforce are not collected on a regular basis, it is challenging to advocate for additional needed resources. Lack of regular enumeration of the public health workforce is an issue facing localities, states, and nations; previous efforts have been limited due to inconsistent definitions including occupational job titles, work settings, and employment status (full-time, part-time, contractual, etc.) as well as differences in methodology [9,10,11].

The New York State Association of County Health Officials (NYSACHO) collaborated with the HRSA-funded Region 2 Public Health Training Center (R2PHTC), a long-time practice partner on the training and technical assistance needs of the public health workforce, to assess the size and composition of New York’s LHD workforce. The goals of this study were to: (1) enumerate NYS’s local public health workforce, (2) assess how the workforce has changed since the COVID-19 pandemic began, and (3) identify challenges to recruitment and retention of qualified public health workers in local health departments. A secondary aim was to use the resulting data to inform state and local policymakers about the potential impacts of budgetary and policy decisions on the delivery of essential public health services.

## 2. Materials and Methods

### 2.1. Study Design

In late 2021, health commissioners and directors or their designees from each LHD in NYS were invited to participate in an online enumeration survey, administered using Qualtrics. Participants were informed that completing the survey was voluntary. The survey collected information about the size of the local public health workforce, including both the number of individuals employed by LHDs and the number of full-time equivalents (FTEs) the employees’ work time comprised in fiscal year 2021. An FTE is defined by the workload, in terms of hours, of a standard employee. Information was also collected on specific occupational titles, the duration of vacancies, reasons for workforce reductions, barriers to hiring, and upcoming retirements. The questionnaire included validated questions from the National Association of City and County Health Officials (NACCHO) 2019 National Profile of Local Health Departments questionnaire [2], to enable longitudinal comparison between the NYSACHO 2021 enumeration data and the NACCHO 2019 data for inferring the impact of COVID-19 on New York’s LHD workforce. Data were requested and received from NACCHO’s 2019 National Profile of LHDs for comparison. Open-ended questions about the main challenges to hiring and retaining staff were also asked. The study was conducted in accordance with the Declaration of Helsinki, and the protocol was approved by Columbia University Irving Medical Center’s institutional review board (Protocol number AAAT0829).

### 2.2. Data Analysis

After the survey responses were collected, we found that three respondents reported implausibly low (zero) values as responses to a question asking for the total number of FTEs currently employed at their LHD. We went back to those three respondents to ascertain corrections, which were provided. Data cleaning involved two imputations: first, answers to the series of questions “Does your LHD currently employ staff in this classification?—<occupational title>” were imputed to “Yes” if the respondent answered the respective question “Please enter the current # of FTE Employed (Filled) Positions in your LHD.—<occupational title>” with a number greater than zero; second, for environmental health titles, missing answers to the series of questions “Does your LHD currently employ staff in this classification?—<occupational title>” were imputed to “No” if the respondent answered the question “Is your LHD partial or full service?” with “Partial Service.” We applied inverse probability weighting in all cross-sectional analyses of the NYSACHO 2021 enumeration data, to adjust for nonparticipation; we conducted sensitivity analyses to assess the magnitude and direction of change, in estimates, due to weighting (Appendix A). We restricted all longitudinal analyses to matched-pair comparisons, using data only for LHDs that provided the respective response in both the NACCHO 2019 survey and the NYSACHO 2021 survey. All data were analyzed using R version 4.2. [12], as well as the tidyverse [13], readxl [14], janitor [15] and scales [16] packages. 

## 3. Results

### 3.1. Participants

Among the 58 LHDs in NYS (Table 1), 89.7% (n = 52) responded to the enumeration survey. The NACCHO 2019 survey collected responses from 63.8% (n = 37) of LHDs in NYS. Both surveys included responses from the New York City Department of Health and Mental Hygiene (NYCDOHMH). Two LHDs responded to the NACCHO 2019 survey, but not to the NYSACHO 2021 survey; 17 LHDs responded to the NYSACHO 2021 survey, but not to the NACCHO 2019 survey; 35 LHDs responded to both surveys; and, 4 LHDs responded to neither survey.

### 3.2. Workforce Size, Type and Composition

LHDs in NYS employed an estimated 11,674 FTEs during 2021: 68.6% (n = 8011) were employed full-time; 17.7% (n = 2063) were employed as contractual staff; 13.0% (n = 1515) were employed part-time; and, 0.7% (n = 85) were employed as seasonal employees. Nearly two thirds (59.7%) of the FTEs were employed by the NYCDOHMH.

LHDs in NYS employed a weighted mean of 138.1 FTEs in full-time positions, 26.1 FTEs in part-time positions, 35.6 FTEs in contractual positions, and 1.5 FTEs in seasonal positions. However, the number of FTEs employed varied widely by the size of the population served by the LHD. The NYCDOHMH, for example, employed 4091.9 FTEs in full-time positions, 1210.6 FTEs in part-time positions, 1666 FTEs in contractual positions, and 0 FTEs in seasonal positions. For full-time positions, the weighted mean numbers of FTEs employed varied directly with the size of population served category and were higher among urban/suburban LHDs (excluding NYC) than among rural LHDs (Table 2).

**Figure 1:**
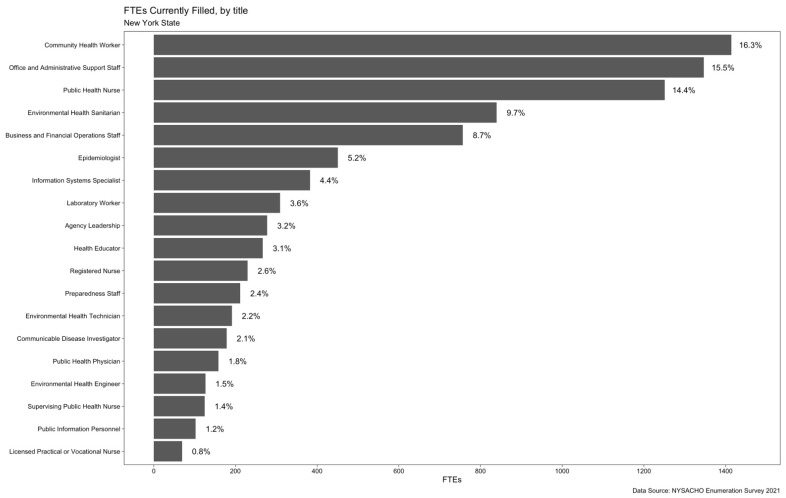
Occupational-Title Composition of the LHD Workforce in NYS (2021). Inverse probability weighting is applied to adjust for nonparticipation.

Community health workers (n = 1414.2), office and administrative support staff (n = 1347.0), and public health nurses (n = 1250.9) comprised nearly half (46.2%) of the FTEs that the responding LHDs employed (Figure 1). In contrast to NYC, in which community health workers filled the plurality share (19.5%) of FTEs, among LHDs outside of NYC office and administrative support staff filled the plurality share (18.2%).

### 3.3. Changes over Time

Among the 34 LHDs that participated in both the 2019 NACCHO survey and the NYSACHO 2021 enumeration survey, the total number of FTEs employed was relatively unchanged, increasing by only 1.7% from 8960 in 2019 to 9111 in 2021. Among the LHDs that responded to both surveys, the total number of FTEs employed at: the NYCDOHMH increased by less than 1%; at a LHD outside of NYC serving an extra-large population (n = 1 LHD) increased by 83%; at LHDs serving large populations (n = 5 LHDs) decreased by 22%; at LHDs serving medium sized populations (n = 15 LHDs) increased by 5%; and, at LHDs serving small populations (n = 14) increased by 12% (Table 3). At the LHD outside of NYC serving an extra-large population, the 83% increase in employed FTEs was primarily attributable to the hiring of contractual staff; full-time FTEs decreased by 55 (from 265 to 210), part-time FTEs increased by 7 (from 123 to 130), contractual FTEs increased by 209 (from 0 to 209), and seasonal FTEs held constant (at 0). The wide range between the percent change in total FTEs employed from 2019 to 2022 for extra-large (+83%) and large (−22%) county sizes can be partially explained by the limited number of counties in those size categories that completed both the NACCHO 2019 and NYSACHO 2021 surveys. While 3 LHDs serving extra-large counties and 8 LHDs serving large counties completed NYSACHO’s 2021 survey, only 1 extra-large and 5 large counties completed NACCHO’s 2019 survey, limiting the comparisons that could be made in this analysis. Future research may be needed to further explore the variation in these changes.

Among the 15 LHDs that were reported, in both the 2019 NACCHO survey and the NYSACHO 2021 Enumeration Survey, the employee types that comprised all employed FTEs, the proportional changes from 2019 to 2021 in the number of FTEs employed varied by employee type. While full-time, part-time, and seasonal FTEs employed decreased by 26%, 9%, and 45%, respectively, the number of contractual FTEs increased by 12,210%, from 13.7 FTEs in 2019 to 1686.0 FTEs in 2021 (Table 4). This was partly attributable to the COVID-19 pandemic response, during which the State and Federal governments provided funds to LHDs to hire contracted staff to assist with COVID-19 mitigation activities such as case-investigation, contact tracing, and vaccination. During the same time, there was a substantial decline in the total number of full-time and part-time FTEs employed.

### 3.4. Workforce Vacancies

Among the 34 LHDs that responded to both the NACCHO 2019 survey and the NYSACHO 2021 survey: 1237.6 FTEs were vacant during 2019, out of 10,198 FTEs budgeted, corresponding with a vacancy rate of 12.1%; 2207.1 FTEs were vacant during 2021, out of 11,318.4 FTEs budgeted, corresponding with a vacancy rate of 19.5%. This comparison suggests that the vacancy rate of LHDs increased statewide between 2019 and 2021. (Table 5) Notably, the vacancy rate at the NYCDOHMH increased from 14.2% (1154 vacant FTEs out of 8110 budgeted FTEs) in 2019 to 21.6% (1925 vacant FTEs out of 8893 budgeted FTEs) in 2021.

**Figure 2:**
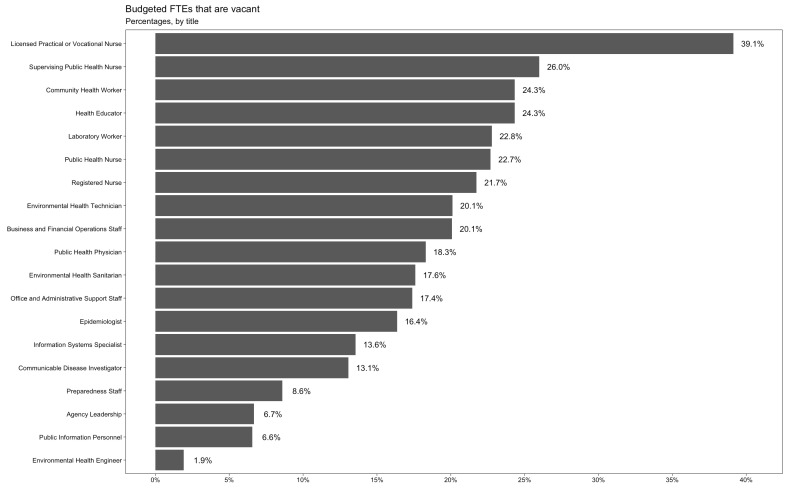
Budgeted FTEs that are Vacant, by Title (2021). Inverse probability weighting is applied to adjust for nonparticipation.

The occupational titles with the highest vacancy rates in 2021 were licensed practical or vocational nurse (39.1%), supervising public health nurse (26.0%), community health worker (24.3%), health educator (24.3%), laboratory worker (22.8%), and public health nurse (22.7%) (Figure 2).

**Figure 3:**
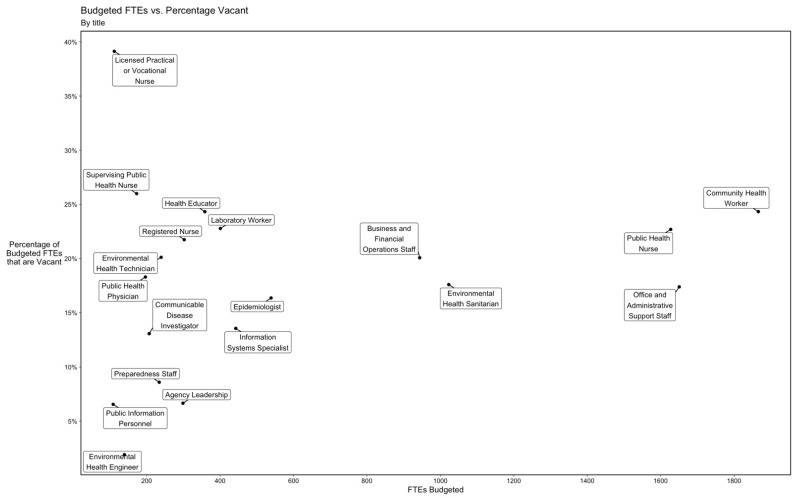
Budgeted FTEs vs. Vacancy Rate Among LHDs in NYS (2021), by Occupational Title. Inverse probability weighting is applied to adjust for nonparticipation.

Statewide, community health workers, public health nurses, and office and administrative support staff had both high numbers of budgeted FTEs and high vacancy rates (Figure 3). Among the LHDs that reported having vacancies in public health nurse positions, a plurality specified that the average length of time those positions were vacant was “more than 12 months” rather than 12 months or less. Collectively, among the estimated 11,553 individuals employed by LHDs in NYS, an estimated 1066 (9.2%) planned to retire within 3 years.

### 3.5. RecruitmentChallenges

When asked about barriers to filling vacancies in specific titles, commissioners or public health directors noted that: (1) salaries are too low to be competitive for positions such as nurses, public health physicians, office and administrative support staff, lab workers, epidemiologists, and others; (2) qualified candidates are hard to find for various roles including health educators, environmental sanitarians and technicians, and community health workers; and, in open-ended responses that (3) the hiring process is too lengthy for candidates to endure due to various administrative hurdles including civil service rules. For example, one respondent reported: “Finding qualified candidates and the length of time to hire due to the Civil Service rules/hiring requirements. The cycle time is very long, average 30 to 60 days to onboard a new employee.” Another respondent reported: “[T]he other difficulty that we have is civil service. Hiring from the list is difficult. We hire people, then have to wait months if not over a year to have a test be offered and then we wait weeks/months for a list to be given to us to hire from. The process is very difficult.”

## 4. Discussion

### 4.1. Sufficient Number of Staff

The decrease in staff between 2019 and 2021 when LHDs were facing the most disruptive public health event in a century is troubling. Data from the NYS Department of Health (NYSDOH) demonstrated that this decrease has been going on for some time. According to NYSDOH, between 2015 and 2020, the number of LHD staff providing Article 6 services decreased by 7% at a time that the state’s population increased by 3% [17]. Similar trends have also been found on a national level. According to data from the Association of State and Territorial Health Officials (ASTHO), from 2012 to 2019, across the United States (US), the state-level health public health workforce decreased by almost 10%, equivalent to an average decrease of 10 FTE public health workers per 100,000 residents. The state-level public health workforce in the US also faces high levels of upcoming retirements, with nearly half (47%) of state health department staff, nationally, reporting that they were considering retiring or leaving their agency for another reason in 2017 [3].

On a global level, varying definitions of public health across countries and regions make it difficult to fully enumerate the global public health workforce. However, a recent Lancet report found that the global health workforce (including physicians, nurses and midwives, dentistry personnel, and pharmaceutical personnel) fell short by approximately 43 million in 2019 [3]. Challenges recruiting staff to the public health sector were discussed in research from Ireland, Portugal, and across Europe. In Ireland, after a recession period from 2008 to 2014, the country’s health workforce grew within acute settings at a much faster rate (33.5%), than within community settings (18.2%). This gap was only exacerbated by COVID-19, and by August 2021, the gap had tripled with almost 25% more FTEs working in acute settings compared to community settings [18]. In the United Kingdom (UK) and European Union (EU), the share of health spending devoted to public health services is small and continues to decline, leading to workforce shortages. Portugal faces the challenge of a rapidly aging public health workforce, with almost 90% of public health doctors being over the age of 50 in 2011 and difficulties recruiting younger people to the field [3].

In 2021, the de Beaumont Foundation and the Public Health Center for Innovations developed a methodology to predict how many additional staff would be needed to provide a minimum package of foundational public health services in LHDs. According to this methodology, 90% of the state’s LHDs did not have the minimum number of staff needed to provide core public health services in their respective communities, and an additional 1000 full-time LHD staff are needed statewide to achieve the minimum level of staffing required [17]. The increase in contractual staff in response to the COVID pandemic may have helped address this gap in staff in the short term, but these temporary staff are dedicated only to one service, and do not fill gaps in staffing needed to provide the core set of public health services every community needs.

While the principal causes of workforce shortages differ by region, country, state, and locality, many of the core sources, identified across existing literature, reflect findings from this study, including low salaries, gaps in training and education, lack of long-term career growth opportunities, and long recruitment timelines [3]. Several European countries, including France, Germany, the Republic of Moldova, Italy, and England report difficulties recruiting students into public health due to low salaries [19]. In England, a lack of specific training and professional development pathways create a barrier to entering the public health field as a long-term career trajectory. As opposed to physicians who have a strict regulatory framework and training pathway, people come to public health from a variety of backgrounds, often having some specialist knowledge, but lacking the formal training needed to assume positions of leadership, a challenge that is shared in the US public health workforce. In Australia, while public health salaries are typically higher, the majority of public health positions (62% in 2018) are for fixed-term contracts and therefore non-permanent positions. Similar to the influx of contractual hires in NYS’s LHDs to assist with COVID-19 response efforts, this model of hiring has implications for the long-term sustainability of the Australian public health workforce [20].

### 4.2. Composition of Staff

Nurses and office- and administrative-support staff comprise a similar proportion of the LHD workforce in NYS as they do nationally [12]. With the exception of an increase in community health workers, a shift reflected nationally where the number of state-level behavioral health professionals increased by 11% between 2012 and 2019, likely due to an increased focus on addressing the opioid crisis, the proportional composition by occupational title of filled positions among LHDs in NYS in 2021 was similar to the composition in 2006, when nurses and support staff comprised substantial proportions of all LHD employees [21,22]. Is this composition still ideal for meeting the responsibilities and priorities that LHDs now face? Conducting routine enumerations using standard definitions and methodologies would enable LHDs in NYS and elsewhere to better understand how the composition of the workforce is meeting those needs.

As new public health threats emerge, demand for skilled workers to perform increasingly complex public health roles is growing, requiring a robust, well-trained, and sustainable public health workforce. However, there currently are no widely adopted standards for public health staffing per capita, nor recommendations for the ideal occupational-title composition of the local public health workforce. Similar issues exist in Europe and the UK, where standards and training for the public health workforce are variable and inconsistent. Partnerships between academic programs, employers, professional organizations, and other stakeholders can help to ensure compatibility between public health training and education programs and workforce needs. Previous successes of these types of partnerships include the strengthening of public health within curricula for nursing, midwifery and environmental health training [23]. Furthermore, these types of partnerships could also be used to develop a set of standards and recommendations for public health staffing.

Such standards and recommendations, especially if developed through a rigorous process and endorsed by national and international public health organizations, could be used widely to guide resource planning and hiring strategy for local health departments; they also could inform advocacy for filling vacancies. However, if such standards or recommendations were to be overly specific, they could hinder LHDs’ abilities to make staffing decisions based on the unique needs of the communities they serve. Further research is needed on potential functions, implications, and content of LHD-staffing standards and recommendations.

### 4.3. Applicability, Strengths, and Limitations of the Survey

This agency-specific survey is a widely applicable tool. Using this tool to enumerate and describe the workforce helps NYS understand its strengths and deficits. This tool could be used by others to help assess their state and local health department’s capacity to meet the needs of their communities and prepare for global responses to our greatest health threats. Potential improvements to the tool could include additional equity and demographic questions to assess how well the workforce represents the communities being served, as well as the knowledge and skills of the workforce.

Their ability to be compared longitudinally with the NACCHO 2019 survey data is a strength of the NYSACHO 2021 survey data. This comparability was a result of similar data collection methods being used for both surveys. In addition to containing shared questions, both surveys were deployed online, and were completed by health commissioners and directors or their designees from LHDs in NYS.

This analysis had limitations. Among the 58 LHDs in NYS, 6 did not respond to the NYSACHO 2021 survey. Additionally, the list of occupational titles included in the survey may not have been exhaustive; furthermore, the occupational titles used in NYS may differ from the ones used in other localities. A potential limitation of the longitudinal analyses was estimate instability due to small sample sizes (especially, for example, after stratification).

Another limitation of this study was the potential for respondents to have different interpretations of employee types (as presented in Table 4)—specifically for contractual employment. The employee-type definitions used in the NYSACHO 2021 survey were taken from the NACCHO 2019 survey to enable longitudinal comparison. A contractual employee was defined in both surveys as “an individual who commits to working for a specified amount of time, and employment will end upon completion of a project or assignment.” However, the survey instructions did not specify whether full-time, part-time, contractual, and seasonal FTEs were mutually exclusive. For example, if interpreted as mutually unexclusive, the FTEs of contracted employees who work full-time hours would be counted as both contractual FTEs and full-time FTEs; in contrast, if interpreted as mutually exclusive, two respondents could have classified those same FTEs differently (one as contractual, and the other as full time). Therefore, the FTEs reported in Table 4 could have been attributed to one or multiple employee types, classified as different employee types by different respondents, or attributed or classified differently in 2019 than in 2021. This potential for different interpretations could have contributed to the large increase in contractual FTEs employed from 2019 to 2021 while the number of full-time FTEs employed decreased.

These findings represent workforce patterns in NYS, but are not necessarily generalizable to other settings. Although the challenges that we found in NYS may differ from those elsewhere, existing literature shows that similar challenges to recruiting and retaining a robust, skilled public health workforce may exist nationally and internationally [2,18,21]. Therefore, we recommend that assessments of LHD workforce challenges outside of NYS consider these findings alongside other common challenges, such as lack of long-term positions, insufficient education pathways, and others cited in this paper.

## 5. Conclusions

This enumeration study found that LHDS across NYS have experienced a decline in the number of full-time staff, and an increase in vacancy rates. Survey respondents reported that inadequate salaries contributed to recruitment and retention challenges within their LHDs. Results from this study have helped NYSACHO advocate for much needed additional funding for NYS’s LHDs. The NYS fiscal year 2022–2023 budget included substantial new funding for LHDs in the form of an increase to their Article 6 base grants, and removed a major barrier which had previously prevented LHDs from using State funding to pay fringe benefits for their staff. These actions should help LHDs to increase their staffing and improve retention. However, without long-term investment in the LHD workforce, gains made this year will not last. Federal, state, and local attention and investments are needed across New York and the United States to bolster the local public health workforce and ensure the health of communities.

## Figures and Tables

**Table 1:** All LHDs in NYS vs. LHDs that Completed the NYSACHO 2021 Enumeration Survey.

	LHDs in NYS	NACCHO 2019 Survey Participants	NYSACHO 2021 Survey Participants
Size of Population Served			
NYC (>2 million)	1	1	1
Extra Large (500,000–2 million)	5	3	3
Large (250,000–499,999)	8	5	8
Medium (75,001–249,999)	18	13	17
Small (<75,000)	26	15	23
Service Level			
Full Service LHDs	36	24	32
Partial Service LHDs	22	13	20
Urbanicity			
NYC	1	1	1
Urban/Suburban (w/o NYC)	25	16	22
Rural	32	20	29
Region			
Capital Region	8	5	8
Central New York	5	5	5
Finger Lakes	9	7	7
Long Island	2	1	1
Mid-Hudson	7	3	6
Mohawk Valley	6	3	5
New York City	1	1	1
North Country	7	5	7
Southern Tier	8	4	7
Western New York	5	3	5

**Table 2:** Weighted Mean (Min-Max) Number of FTEs Employed, by Employee Type, LHDs in NYS (2021).

	Full Time	Part Time	Contractual	Seasonal
Overall	138.1 (6–4092)	26.1 (0–1211)	35.6 (0–1666)	1.5 (0–38)
Size of Population Served				
NYC (>2 million)	4091.9	1210.6	1666.0	0
Extra Large (500,000–2 million)	338.7 (210–585)	25.0 (1–65)	69.7 (0–209)	0.0 (0–0)
Large (200,000–499,999)	101.2 (30–163)	4.6 (0–13)	0.4 (0–1)	6.2 (0–38)
Medium (75,001–199,999)	45.8 (10–89)	2.7 (0–18)	1.2 (0–8)	1.2 (0–21)
Small (<75,000)	22.8 (6–87)	3.6 (0–40)	0.9 (0–5)	0.5 (0–10)
Urbanicity				
Urban/Suburban (excl. NYC)	125.8 (16–585)	8.3 (0–65)	14.6 (0–209)	2.9 (0–38)
Rural	25.3 (6–87)	3.1 (0–40)	1.2 (0–8)	0.4 (0–10)

Inverse probability weighting is applied to adjust for nonparticipation.

**Table 3:** Total FTEs Employed in LHDs, NYS (2019 vs. 2021).

	NACCHO 2019	NYSACHO 2021	Difference	% Change	n
Overall					
All Respondents	8960	9111	151	2%	34
Size of Population Served					
NYC (>2 million)	6956	6968	12	0%	1
Extra Large (500,000–2 million)	265	484	219	83%	1
Large (200,000–499,999)	721	561	−160	−22%	5
Medium (75,001–199,999)	612	643	31	5%	13
Small (<75,000)	407	455	49	12%	14

Data are restricted to only from LHDs that provided this information on both surveys. Includes FTEs of full-time, part-time, contractual, and seasonal employees.

**Table 4:** Total FTEs Employed in LHDs (n = 15), by Employee Type, NYS (2019 vs. 2021).

Employee Type	NACCHO 2019	NYSACHO 2021	Difference	% Change
Full Time	6428.0	4751.0	−1677.4	−26%
Part Time	1347.6	1225.0	−123.0	−9%
Contractual	13.7	1686.0	1672.8	12,210%
Seasonal	58.4	32.0	−26.4	−45%

Data are restricted to only from LHDs (n = 15) that provided this information on both surveys.

**Table 5:** Vacancy Rates Among LHDs in NYS (2019 vs. 2021).

		Budgeted			Vacant			Vacancy Rate		
Population	n	2019	2021	Change	2019	2021	Change	2019	2021	Change
All Respondents	34	10,198.00	11,318.40	1120.40	1237.60	2207.10	969.5	12.10%	19.50%	7.40%
NYC (>2 million)	1	8110.00	8893.10	783.1	1154.00	1924.70	770.7	14.20%	21.60%	7.40%
Extra Large (500,000–2 million)	1	281	520	239	16	36	20	5.70%	6.90%	1.20%
Large (200,000–499,999)	5	755.5	678	−77.5	34.5	117	82.5	4.60%	17.30%	12.70%
Medium (75,001–199,999)	13	631.7	712.2	80.5	19.9	69.5	49.6	3.20%	9.80%	6.60%
Small (<75,000)	14	419.8	515.1	95.3	13.2	59.9	46.7	3.10%	11.60%	8.50%

Data are restricted to only from LHDs that provided this information on both surveys.

**Figure A1:**
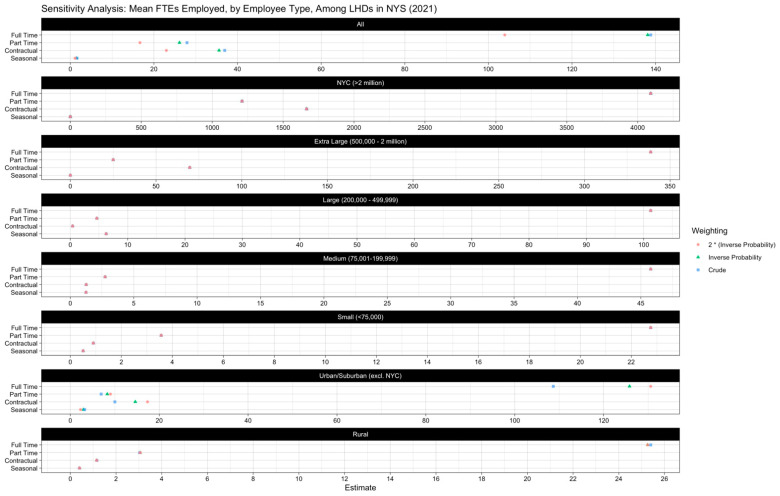
Sensitivity Analysis for Table 2.

**Figure A2:**
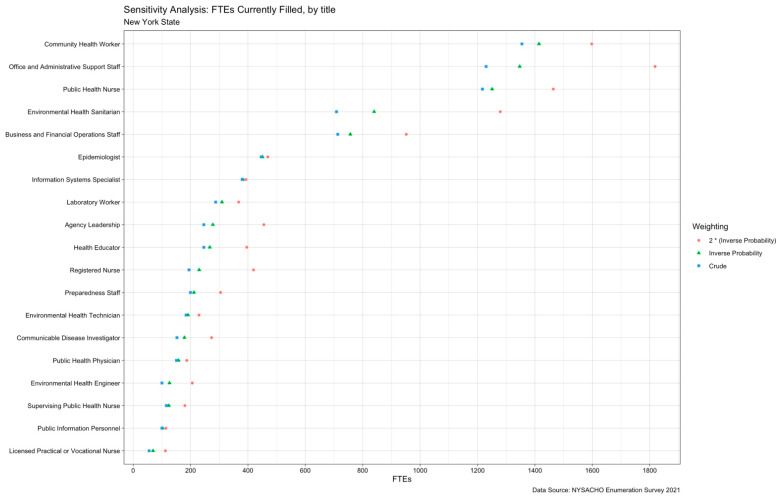
Sensitivity Analysis for Figure 1.

**Figure A3:**
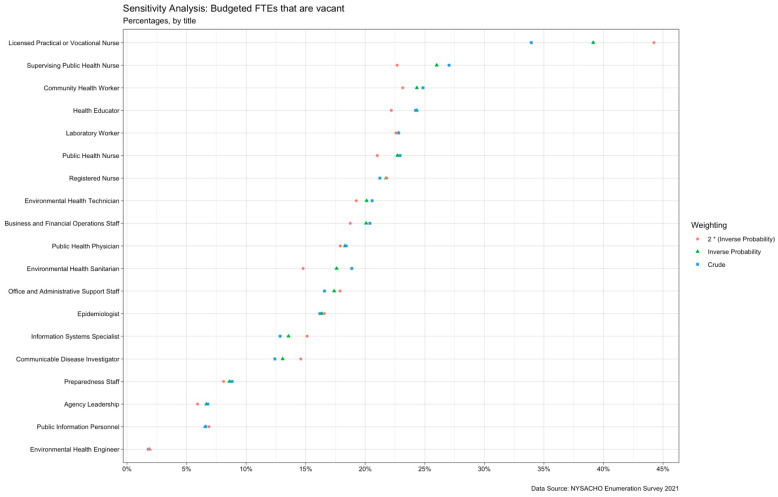
Sensitivity Analysis for Figure 2.

**Figure A4:**
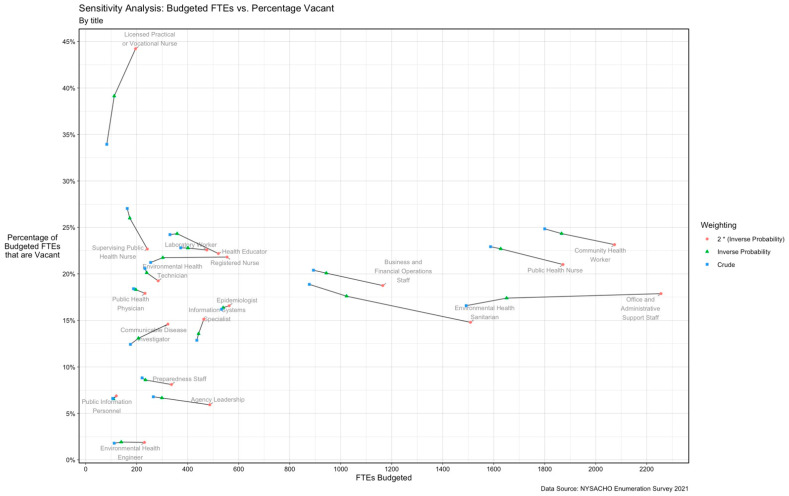
Sensitivity Analysis for Figure 3.

## Data Availability

Not applicable.

## Appendix A

The following figures (Figure A1, Figure A2, Figure A3 and Figure A4) are sensitivity analyses of the cross-sectional analyses reported via Table 2, and Figure 1, Figure 2 and Figure 3. The sensitivity analyses compare, graphically, three variations of each estimate: crude, weighted by inverse probability of selection (with respect to size of the population served by the LHD), and weighted by two times the inverse probability.
